# Supplementary material for: Pathological and immunological evaluation of different regimens of praziquantel treatment in a mouse model of Schistosoma mansoni infection
Source: PLoS Negl Trop Dis. 2022 Apr 21;16(4):e0010382. doi: 10.1371/journal.pntd.0010382 (PMC9064093; doi:10.1371/journal.pntd.0010382)
Supplement: Attachment — Submitted filename: Responses to reviewers_PNTD-D-22-00124.docx [file pntd.0010382.s001.docx]

**Response to reviewers**

**Manuscript PNTD-D-22-00124**

**Manuscript title: Pathological and immunological evaluation of different regimens of praziquantel treatment in a mouse model of *Schistosoma mansoni* infection**

**Key Review Criteria Required for Acceptance**

As you describe the new analyses required for acceptance, please consider the following:

**Methods**

-Are the objectives of the study clearly articulated with a clear testable hypothesis stated?

-Is the study design appropriate to address the stated objectives?

-Is the population clearly described and appropriate for the hypothesis being tested?

-Is the sample size sufficient to ensure adequate power to address the hypothesis being tested?

-Were correct statistical analysis used to support conclusions?

-Are there concerns about ethical or regulatory requirements being met?

Reviewer #1: (No Response)

Reviewer #2: I would prefer a replicated study design for any study of drug effects. However, given the complexity of the current report, I will not insist on it. I am concerned that pretesting of the dose regimens was not performed prior to treatment, as extended administration of 100 mg/kg PZQ is clearly toxic; these regimens should not have been included in experiments with infected animals. I have not checked, but surely some data on toxicology were available; the package submitted for approval would have included results from chronic testing and should be available from WHO, if not other sources. The authors should discuss this shortcoming. Is it possible that infection exacerbated the toxicity of PZQ?

**Answer: According to our results, we could not assess that *S. mansoni* infection exacerbated the toxicity of PZQ in infected mice treated with PZQ at 100 mg/kg/day for 28 consecutive days because the infection was practically cleared in mice of this group.**

In addition, the authors should have included a group treated with the standard regimen of PZQ in their model. Some authors treat with a single dose of up to 500 mg/kg at 28 days, others at 42 days.

**Answer: In this study, we did not intend to treat schistosomiasis when the parasite is already adults (standard regimen of 500 mg/kg of PZQ at 28 or 42 days), but we focused on the juvenile stages. Thus, we aimed to evaluate the effect of praziquantel regimens early administration on the parasite growth and maturation and its outcomes during the granulomatous stage of the infection in mice. We, therefore, used four PZQ regimens, among which a single dose of 500 mg/kg but at day one post-infection since authors have demonstrated that PZQ is efficient on early young schistosomula (PMID: 15013742; PMID: 20627145).**

**Results**

-Does the analysis presented match the analysis plan?

-Are the results clearly and completely presented?

-Are the figures (Tables, Images) of sufficient quality for clarity?

Reviewer #1: (No Response)

Reviewer #2: No concerns about this aspect of the manuscript

--------------------

**Conclusions**

-Are the conclusions supported by the data presented?

-Are the limitations of analysis clearly described?

-Do the authors discuss how these data can be helpful to advance our understanding of the topic under study?

-Is public health relevance addressed?

Reviewer #1: (No Response)

Reviewer #2: (No Response)

--------------------

**Editorial and Data Presentation Modifications?**

Use this section for editorial suggestions as well as relatively minor modifications of existing data that would enhance clarity. If the only modifications needed are minor and/or editorial, you may wish to recommend “Minor Revision” or “Accept”.

Reviewer #1: (No Response)

Reviewer #2: (No Response)

--------------------

**Summary and General Comments**

Use this section to provide overall comments, discuss strengths/weaknesses of the study, novelty, significance, general execution and scholarship. You may also include additional comments for the author, including concerns about dual publication, research ethics, or publication ethics. If requesting major revision, please articulate the new experiments that are needed.

Reviewer #1: This was an interesting study of the effects of praziquantel on both the schistosome parasites and the host. The work is comprehensive and well performed. The manuscript is very easy to real and all of the figures of a high standard. There is a huge amount of data presented. While this makes the study very comprehensive, it does make the publication unwieldly as a whole.

Line 107 please state the sex of the animals used.

**Answer: Added as suggested (please see line 107)**

I would be useful in groups could be named better ie HC, IC PZQ1-4 etc at the beginning of the line in the methods section.

**Answer: Change made as suggested (please see from lines 117 to 126)**

The use of PZQ dissolved in distilled water is a concern, do the drugs solubility. The FDA states it is “and very slightly soluble in water”

<https://www.accessdata.fda.gov/drugsatfda_docs/label/2014/018714s013lbl.pdf>

**Answer: According to the document given in the above link, it is the pure powder of praziquantel that is slightly soluble in water. In our experimentation, we used a praziquantel tablet (Cesol®) soluble in water. During the preparation of the PZQ solution, we made a vigorous agitation to ensure that all the PZQ tablet was dissolved in water as mentioned by others (PMID:34280206).**

Just to confirm the third group received 100mg/kg/day x 28 days, that contrasts to the other groups that in total received 500mg/kg, that 3rd group received 5 times the dosage of the others. I am not sure how that was justified, especially in the light of the very low survival rate.

**Answer: We set up this group to assess the toxicity of praziquantel as in a case of subchronic administration.**

In Table 2 define what the acute and granulomatous phases are.

**Answer: Change made as suggested (please see lines 303 and 304)**

Similarly in line 387 the terms acute stage is used but not defined until line 532. Same with granulomatous stage on line 291.

**Answer: Change made as suggested (please see lines 290 and 390, respectively)**

Table 1 please add references for the primers used if possible.

**Answer: Added as suggested: “all primers were synthesized according to the PrimerBank online database”.** [**https://pga.mgh.harvard.edu/primerbank/citation.html**](https://pga.mgh.harvard.edu/primerbank/citation.html) **(please see lines 236 and 237)**

Tables 3-5 were cut off on the right in the pdf I reviewed. I could not review these properly. I am not sure why this was not identified during the submission process.

**Answer: Please, regarding this inconvenience, we followed the recommendations prescribed by the journal submission guideline: “***Place each table in your manuscript file directly after the paragraph in which it is first cited (read order). Do not submit your tables in separate files****.*** *Tables do not have strict width and height requirements. Do not split your table or otherwise try to make the table appear within the manuscript margins if it does not fit on one page. In Word, tables that run off of the manuscript page can be seen using Draft View. In the PDF version of the published article, very wide tables may be printed sideways, and long tables may span more than one page”****.***

**However, we arranged it on the revised version of the manuscript.**

From what I can is that AST levels were elevated for the PZQ3 group? Since there were no groups where uninfected mince were given the PZQ regimes tested, there is no way to demonstrated the cytotoxic effects for the drug on the animal. But I acknowledge that the group PZQ 3 parameters are less than IC. The long term (28 days) exposure of cell lines to PZQ could have been informative.

**Answer: Thank you for this idea. We will plan this as an upcoming study.**

The efficacy of a low dosage for a long period of time is clear in the many metrics provided, but what is the potential for drug resistance to develop in field settings? Drug resistance in general is not discussed.

While the results are very robust, it is not clear to me if the authors are saying if PZQ is having an effect on the host, independent to its direct antischistosomal activity. There have been some reports on the therapeutic effect of PZQ (PMID: 32606340). I would recommend these papers be considered for your manuscript. PMID:34736899, PMID:34796025, PMID:34702326, PMID:34273315, PMID:34280206, PMID: 32741402, PMID:23555262.

**Answer: Thank you for your suggestion; we considered two of them (PMID: 32606340 and PMID:34280206)** **and inserted them in the manuscript**

Reviewer #2: The authors should discuss the possibility that PZQ bioaccumulates in chronic treatment, so that its normally short t-1/2 is extended after 28 days of dosing, especially if liver damage is present.

**Answer: Thank you for this suggestion. Considering the short t-1/2 (0.8 to 1.2 hours), we think that the liver damage observed could result from a daily cumulative toxic effect of 100 mg/kg of PZQ after 28 days of treatment.**

Efficacy of 28-day regimens could be due to sufficient presence of the drug to affect worms of sufficient maturity at day 28 to be susceptible, or to previously unknown effects of prolonged duration of exposure to PZQ on immature worms. Without data on plasma levels at day 28/29, these possibilities cannot be rigorously excluded.

Also, while it is true that it is desirable to prevent egg deposition in the host, daily dosing is incompatible with current control programs in the field; to prevent the development of adult parasites, people would have to receive a month of treatment every other month. It is possible that expatriates or tourists could be given a month-ling regimen of PZQ (assuming these results are relevant for humans), but it is also possible to take a normal regimen 6 weeks after leaving an endemic area to prevent pathology.

The results are interesting and should be published, as they indicate that an extended duration of exposure, independent of the dose (and thus plasma levels) may be deleterious to immature schistosomes. That, I believe, is new.

**Answer:** **Thank you for your review. I am fill honoured to receive your appreciation and your valuable suggestions and comments.**
